# Age-related accrual of methylomic variability is linked to fundamental ageing mechanisms

**DOI:** 10.1186/s13059-016-1053-6

**Published:** 2016-09-22

**Authors:** Roderick C. Slieker, Maarten van Iterson, René Luijk, Marian Beekman, Daria V. Zhernakova, Matthijs H. Moed, Hailiang Mei, Michiel van Galen, Patrick Deelen, Marc Jan Bonder, Alexandra Zhernakova, André G. Uitterlinden, Ettje F. Tigchelaar, Coen D. A. Stehouwer, Casper G. Schalkwijk, Carla J. H. van der Kallen, Albert Hofman, Diana van Heemst, Eco J. de Geus, Jenny van Dongen, Joris Deelen, Leonard H. van den Berg, Joyce van Meurs, Rick Jansen, Peter A. C. ‘t Hoen, Lude Franke, Cisca Wijmenga, Jan H. Veldink, Morris A. Swertz, Marleen M. J. van Greevenbroek, Cornelia M. van Duijn, Dorret I. Boomsma, BIOS consortium, P. Eline Slagboom, Bastiaan T. Heijmans

**Affiliations:** 1 Molecular Epidemiology section, Leiden University Medical Center, Einthovenweg 20, 2333 ZC Leiden, The Netherlands; 2 Department of Genetics, University Medical Centre Groningen, Antonius Deusinglaan 1, 9713 AV Groningen, The Netherlands; 3 Sequence Analysis Support Core, Leiden University Medical Center, Einthovenweg 20, 2333 ZC Leiden, The Netherlands; 4 Department of Human Genetics, Leiden University Medical Center, Einthovenweg 20, 2333 ZC Leiden, The Netherlands; 5 Department of Internal Medicine, Erasmus University Medical Center, Wytemaweg 80, 3015 CN Rotterdam, The Netherlands; 6 Department of Internal Medicine and School for Cardiovascular Diseases (CARIM), Maastricht University Medical Center, Universiteitssingel 50, 6229 ER Maastricht, The Netherlands; 7 Department of Epidemiology, Erasmus University Medical Center, Dr. Molewaterplein 50, 3015 GE Rotterdam, The Netherlands; 8 Department of Gerontology and Geriatrics, Leiden University Medical Center, Albinusdreef 2, 2333 ZA Leiden, The Netherlands; 9 Department of Biological Psychology, VU University Amsterdam, Neuroscience Campus Amsterdam, Van der Boechorststraat 1, 1081 BT Amsterdam, The Netherlands; 10 Department of Neurology, Brain Center Rudolf Magnus, University Medical Center Utrecht, Universiteitsweg 100, 3584 CG Utrecht, The Netherlands; 11 Department of Psychiatry, VU University Medical Center, Neuroscience Campus Amsterdam, A.J. Ernststraat 1187, 1081 HL Amsterdam, The Netherlands; 12 Genomics Coordination Center, University Medical Center Groningen, University of Groningen, Hanzeplein 1, 9713 GZ Groningen, The Netherlands; 13 Department of Genetic Epidemiology, Erasmus University Medical Center, Dr. Molewaterplein 50, 3015 GE Rotterdam, The Netherlands

**Keywords:** DNA methylation, Ageing, 450k, DNA damage, Variability

## Abstract

**Background:**

Epigenetic change is a hallmark of ageing but its link to ageing mechanisms in humans remains poorly understood. While DNA methylation at many CpG sites closely tracks chronological age, DNA methylation changes relevant to biological age are expected to gradually dissociate from chronological age, mirroring the increased heterogeneity in health status at older ages.

**Results:**

Here, we report on the large-scale identification of 6366 age-related variably methylated positions (aVMPs) identified in 3295 whole blood DNA methylation profiles, 2044 of which have a matching RNA-seq gene expression profile. aVMPs are enriched at polycomb repressed regions and, accordingly, methylation at those positions is associated with the expression of genes encoding components of polycomb repressive complex 2 (PRC2) *in trans*. Further analysis revealed *trans*-associations for 1816 aVMPs with an additional 854 genes. These *trans*-associated aVMPs are characterized by either an age-related gain of methylation at CpG islands marked by PRC2 or a loss of methylation at enhancers. This distinct pattern extends to other tissues and multiple cancer types. Finally, genes associated with aVMPs *in trans* whose expression is variably upregulated with age (733 genes) play a key role in DNA repair and apoptosis, whereas downregulated aVMP-associated genes (121 genes) are mapped to defined pathways in cellular metabolism.

**Conclusions:**

Our results link age-related changes in DNA methylation to fundamental mechanisms that are thought to drive human ageing.

**Electronic supplementary material:**

The online version of this article (doi:10.1186/s13059-016-1053-6) contains supplementary material, which is available to authorized users.

## Background

Studies of model organisms such as yeast, nematodes, and mice have shown that the accumulation of cellular damage is a fundamental cause of ageing across species [1–3]. Epigenetic dysregulation is thought to play a key role in this process [4, 5]. Numerous human population studies have now shown that changes in DNA methylation of CpG dinucleotides, a key epigenetic mechanism [6], are strongly associated with chronological age. Although these epigenetic changes are in part a by-product of age-related changes in the cellular composition of the studied tissue [7–9], many age-related differentially methylated positions (aDMPs) observed in blood samples are independent of cell composition [8–19]. Additional studies showed the consistent occurrence of aDMPs in other tissues [20–22] and species [23] and aDMPs have proven to be a useful tool to predict chronological age [24, 25].

However, aDMPs may not be the most informative marker of the ageing process since they were discovered as close correlates of chronological age instead of biological age [26]. Moreover, only a small proportion of aDMPs are associated with expression changes [12, 17], suggesting that their functional implication may be limited. In contrast, DNA methylation changes that increasingly diverge from chronological age may reflect the increasing inter-individual variation in health that occurs with increasing age. Initial studies, although small or lacking a genome-wide view, indicated that an increasing variability of DNA methylation with age indeed exists [21, 27, 28]. A recent large twin study showed that genetic factors explain a minor proportion of the variation in DNA methylation in the population, while the influence of environmental and stochastic factors is large and commonly increases with age [19].

In the current study, we charted the occurrence of age-related variably methylated positions (aVMPs) across the genome. We evaluated the methylation at 429,296 CpG sites for increased variability with age in whole blood samples from 3295 individuals. After validation using multiple external datasets and integration of methylome with transcriptome data (n = 2044), we show that aVMPs link to the expression of genes *in trans* that play a central role in fundamental ageing mechanisms.

## Results

**Fig. 1:**
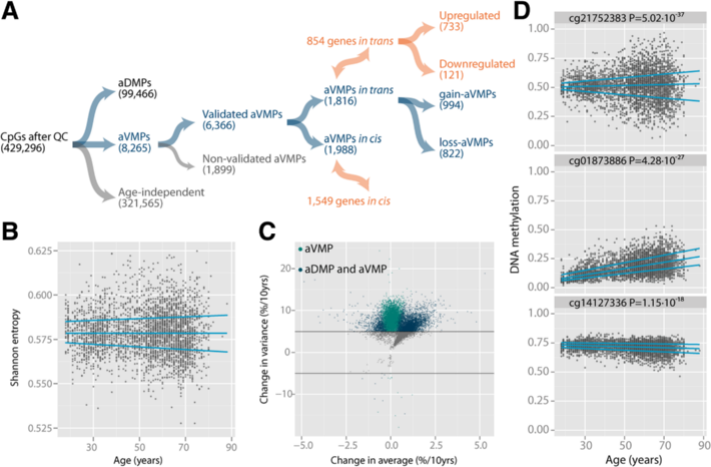
Discovery of aVMPs in whole blood of 3295 individuals. **a** Flow chart of the different sets of CpGs identified in the current study, *in cis* and *in trans. QC* quality control. **b** Shannon entropy (*y-axis*) against age (*x-axis*) in 3295 individuals. **c** Volcano plot of the DNA methylation change in average (*x-axis*) against the change in variability (*y-axis*). **d** Examples of the three classes of aVMPs identified: aVMPs without a change in average (constant-aVMPs, cg21752383), aVMPs with a gain in DNA methylation (gain-aVMPs; cg01873886), and aVMPs with a loss in methylation (loss-aVMPs; cg14127336)

To uncover the occurrence of divergence in DNA methylation with age, we studied 3295 whole-blood DNA methylation profiles encompassing 429,296 CpGs of individuals aged 18 to 88 years (Fig. 1a; Additional file 1a, b). To obtain global evidence for an increasing DNA methylomic divergence between individuals with age, we calculated the Shannon entropy on the individual level [25, 29]. With age, we observed a distinct increase in the variability in the Shannon entropy (adjusted for age, sex, and blood cell composition), suggesting that the level of order between individuals becomes more variable at older ages (Fig. 1b).

To map the specific CpGs that change in variability with age (age-related variably methylated positions or aVMPs), we tested for an increase in variability with age, independent of an average change in DNA methylation with age (aDMP effects) and changes in blood cell composition. We identified 8265 aVMPs that showed a robust increase in variability with age (*P* < 10^−7^ and ≥5 % increase per 10 years; Fig. 1a, c). The number of aVMPs was substantially smaller than aDMPs in our data set: approximately a quarter of the CpGs tested were identified as aDMPs (99,466 CpGs, no effect size cutoff; Fig. 1a; Additional file 1d); the majority of the CpGs did not show an age-related change in DNA methylation (321,565 CpGs; Fig. 1a; Additional file 1c). The number of aVMPs showing a decrease in variability with age was exceedingly small (19 aVMPs, *P* < 10^−7^ and ≥5 % decrease per 10 years) and these aVMPs were excluded from further analyses (Fig. 1c). Since increases in variability at hypo- or hypermethylated CpGs can only go in one direction (up for DNA methylation levels near 0 and down for those near 1), we observed that 48.2 % of the aVMPs were also identified in these data as aDMPs (3980 aVMPs; Fig. 1c). Of note, only 213 aVMPs (2.6 %) overlapped with previously reported aDMPs (n = 7477) [7, 8, 14, 16, 18, 25] and six aVMPs overlapped with CpGs from the Horvath clock (n = 353) [24] (Additional file 1e). Hence, aVMPs represent a new and distinct class of age-related changes in DNA methylation. Clustering analysis showed that aVMPs could be categorized into three groups: aVMPs hypomethylated in young individuals that gained DNA methylation with age (both average and variability; gain-aVMPs), hypermethylated aVMPs that lost DNA methylation with age (both average and variability; loss-aVMPs) and intermediate-methylated aVMPs where gains and losses with age were balanced, resulting in the absence of an aDMP effect (constant-aVMPs) (Fig. 1d).

**Fig. 2:**
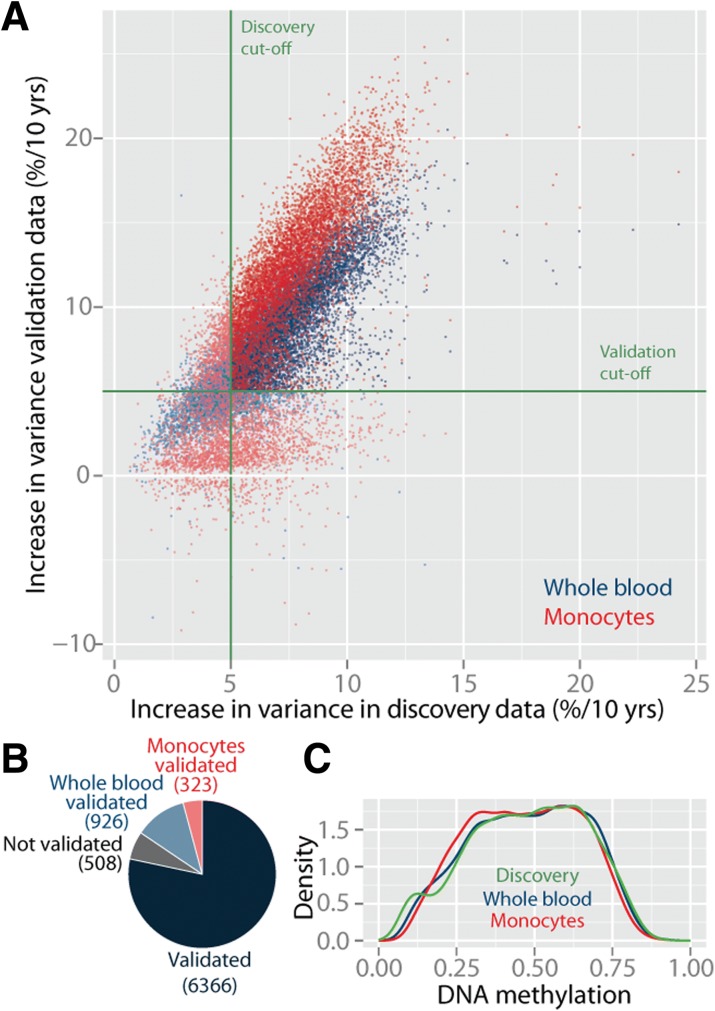
Validation of aVMPs in whole blood of 643 and purified monocytes of 1202 individuals. **a** Change in variability in the discovery (*x-axis*) against the whole blood and monocyte validation (*y-axis*) datasets. *Red dots* represent the monocyte validation dataset, *blue dots* represent the whole blood validation dataset. **b** The aVMPs that validate in whole blood and monocytes. **c** Density plot of average DNA methylation of validated aVMPs in the discovery dataset and validation datasets

We validated the aVMPs in two large public datasets consisting of whole blood (n = 643, age range 20–102, median 65 years) [25] and purified monocytes, a relative homogeneous population of blood cells (n = 1202, age range 44–83, median 59 years) [17]. This validation step was incorporated to exclude that our results were confounded by the fact that we analyzed multiple population studies of different ages or by age-related changes in the cellular composition of blood. The increase in variability observed in the discovery data was remarkably concordant with the effect size in both validation datasets (Fig. 2a). In total, 6366 aVMPs (78.4 %) were validated in both datasets (Fig. 2b; Additional file 2).

Although we corrected for cell heterogeneity using the major blood cell subtypes, age-related changes in the percentage of less frequent cell subtypes could also have introduced aVMPs. To further exclude that the increased variability was driven by changes in blood cell composition, we repeated our aVMP analysis with the inclusion of predicted, more refined blood cell subtype fractions (CD8^+^ T cells, CD4^+^ T cells, natural killer (NK) cells, B cells, and granulocytes) [30]. Out of the 6366, 6343 (99.6 %) were again identified as aVMPs (*P*_*FDR*_ ≤ 0.05). Furthermore, we compared the DNA methylation of whole blood to that of blood cell subtypes and their progenitors using five public data sets on 19 cell types (Additional file 3). The methylation of aVMPs was highly concordant between whole blood and the various cell subtypes, indicating aVMPs cannot be attributed to the outgrowth of a specific cell subtype, but instead represent genuine age-related changes in DNA methylation. Conversely, CpGs differentially methylated between blood cell subtypes [31] had the same, if not smaller, chance of being an aVMP (odds ratio = 0.56; *P* = 0.19).

The validated set of 6366 robust and consistent aVMPs was taken forward for in-depth analysis. aVMPs were characterized by an intermediate methylation level (Fig. 2c). Among the 6366 aVMPs, a similar number of gain-aVMPs and loss-aVMPs were found (2788 gain-aVMPs, 3578 loss-aVMPs). While some individuals showed differential DNA methylation on many of the identified aVMPs (marked by vertical bands in Additional file 1f), others did not show any difference in DNA methylation. The individual-specific changes in DNA methylation with age also resulted in an age-related loss of correlation between individuals on the 6366 aVMPs (Additional file 1g).

### aVMPs are depleted in active regions and enriched in PcG repressed regions

**Fig. 3:**
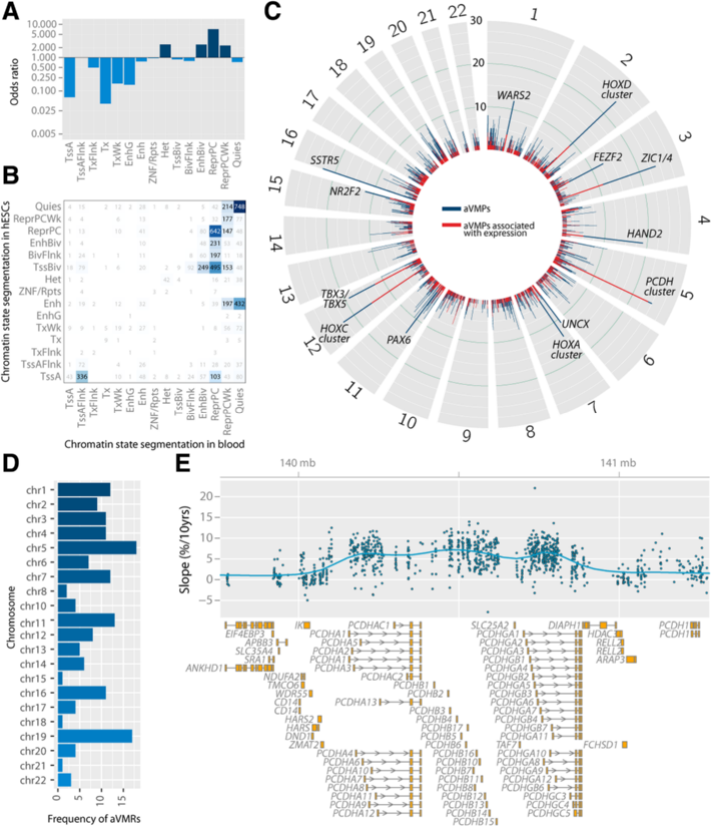
Characterization of genomic regions harboring aVMPs and associations of gene expression *in cis*. **a** Enrichment (odds ratio, *y-axis*) of aVMPs in chromatin state segments (*x-axis*) in blood. **b** Overlap between chromatin state segmentation data in blood and in human embryonic stem cells (*hESCs*). Numbers represent the number of aVMPs that overlap between segments. **c** Frequency of aVMPs (*y-axis*) in 100-kb windows across the genome (*blue bars*) and their association with gene expression *in cis* in *red*. **d** Frequency of age-related variably methylated regions (*aVMRs*; *x-axis*) on each of the autosomal chromosomes (*y-axis*). **e** Increased variability (*y-axis, top panel*) of the protocadherin cluster (*bottom panel*). *Abbreviations*: *TssA* Active transcription start site, *TssAFlnk* flanking active transcription start site, *TxFlnk* transcription at gene 5′ and 3′, *Tx* strong transcription, *TxWk* weak transcription, *EnhG* genic enhancers, *Enh* enhancers, *ZNF/Rpts* ZNF genes plus repeats, *Het* heterochromatin, *TssBiv* bivalent/poised transcription start site, *BivFlnk* flanking bivalent transcription start site/enhancer, *EnhBiv* bivalent enhancer, *ReprPC* repressed polycomb, *ReprPCWk* weak repressed polycomb, *Quies* quiescent/low

To characterize the genomic regions harboring aVMPs, we obtained the chromatin state segments of blood cell types (Epigenomics Roadmap [32]), which reflect the biological function of the underlying region in blood cells on the basis of combinations of histone modifications [32]. A pronounced enrichment was found for aVMPs in the segments marking a repressed genome, including *repressed polycomb* (7.2-fold enrichment, *P* < 0.0001), *weak repressed polycomb* (2.3-fold enrichment, *P* < 0.0001) and *heterochromatin* (2.5-fold enrichment, *P* < 0.0001), while aVMPs were depleted for active segments, including *strong transcription* (0.04-fold enrichment, *P* < 0.0001) (Fig. 3a; Additional file 4b). In absolute terms, 4212 aVMPs (66.2 %) mapped to segments marking repressed DNA. These data were supported by a parallel enrichment of aVMPs for repressive histone modifications (Additional file 4a) and for binding sites of the PcG repressive complex 2 (PRC2) protein EZH2 in the ENCODE blood cell line GM12878 (2.1-fold enrichment, *P <* 0.0001).

To explore whether the methylation level of aVMPs was associated with gene expression *in cis* (i.e., gene and aVMP within 500 kb), we studied the relationship between DNA methylation and gene expression using 2044 individuals for whom both DNA methylation and gene expression data (RNA-seq) were available. Despite predominantly mapping to repressed regions, 1988 *cis*-aVMPs out of the 6366 aVMPs (31.2 %; Fig. 1a; Additional file 5) were associated with gene expression of 1549 genes *in cis* (see Additional file 4c for *HOXC4* and *PCDHB6* examples). Gene Ontology (GO) analysis of the *cis*-associated genes showed enrichment for processes involved in *neuron differentiation* and *neuron development* (*P <* 0.0001).

Since age-related divergence was found to be overrepresented near developmental genes, we also mapped aVMPs to chromatin state segments of a human embryonic stem cell line (H1-hESC). Interestingly, repressed genomic regions in blood harboring an aVMP were often a (bivalent) transcription start site or (bivalent) enhancer in the H1-hESC line (Fig. 3b), highlighting the developmental history of regions accumulating aVMPs.

In line with the enrichment for developmental genes, we found a large number of aVMPs near (neuro)developmental genes (Fig. 3c). To investigate whether aVMPs clustered into regions, we identified age-related variably methylated regions (aVMRs) [33]. This resulted in 160 aVMRs encompassing 527 aVMPs (8.3 %; Additional file 6). aVMRs were particularly frequent on chromosomes 5 and 19 (Fig. 3d), the former of which could be attributed, in part, to eight aVMRs (totalling 26 aVMPs) that mapped to the protocadherin gene cluster (Fig. 3e).

### aVMPs are associated with expression of genes *in trans*

**Fig. 4:**
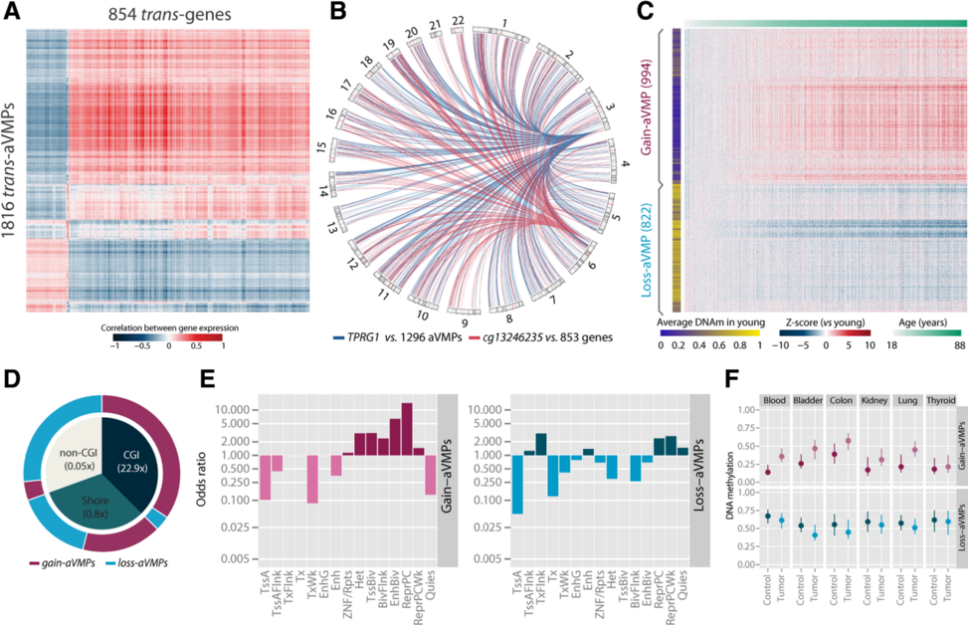
Identification and characterization of aVMPs associated with gene expression *in trans*. **a** Correlation between DNA methylation of 1816 aVMPs (*columns*) and gene expression of 854 genes (*rows*). **b** *TPRG1* is associated with 1296 aVMPs and cg13246235 with the expression of 853 genes. *Blue lines* represent the associations between the gene expression of *TPRG1* and the DNA methylation of 1296 aVMPs. *Red lines* represent the associations between the DNA methylation of cg13246235 and the expression of 853 genes. **c** Z-score of individuals (*columns*) versus young individuals (<30 years) of *trans*-aVMPs (*rows*). The *bar on the bottom left* represents the average DNA methylation (*DNAm*) at a young age (<30 years). The *bar on the top* represents the age from low age (*white*) to high age (green). **d** Fraction and enrichment of gain- and loss-aVMPs in genomic features. *CGI* CpG island. *Blue*, fraction of loss-aVMPs; *purple*, fraction of gain-aVMPs. **e** Enrichment (odds ratio, *y-axis*) of gain- and loss-aVMPs in chromatin state segments (*x-axis*). **f** Average DNA methylation of aVMPs in various cancer types compared with their normal tissue counterpart. *Abbreviations*: *TssA* Active transcription start site, *TssAFlnk* flanking active transcription start site, *TxFlnk* transcription at gene 5' and 3', *Tx* strong transcription, *TxWk* weak transcription, *EnhG* genic enhancers, *Enh* enhancers, *ZNF/Rpts* ZNF genes plus repeats, *Het* heterochromatin, *TssBiv* bivalent/poised transcription start site, *BivFlnk* flanking bivalent transcription start site/enhancer, *EnhBiv* bivalent enhancer, *ReprPC* repressed polycomb, *ReprPCWk* weak repressed polycomb, *Quies* quiescent/low

The enrichment of aVMPs in polycomb-repressed regions and PRC2 binding sites suggests a role for PcG proteins in the occurrence of aVMPs. Indeed, methylation at the majority of aVMPs was positively associated with the expression of components of the PRC2, including *EED*, *SUZ12*, and *EZH2*, particularly when an aVMP mapped to an EZH2 binding site (Additional file 7a). Further analysis revealed that *trans*-associations were not limited to PRC2 components. Of the total number of 6366 aVMPs, 1816 (28.5 %) were associated with expression of 854 coding genes *in trans* (4.6 % of genes tested; Fig. 4a; Additional file 8), i.e., the aVMP and gene on different chromosomes or on the same chromosome but 5 Mb apart. The association between aVMP methylation and gene expression implies an increase in variance in gene expression in conjunction with DNA methylation. Of note, the number of associations between *trans*-genes and *trans*-aVMPs was high. For example, the expression of *TPRG1* was associated with 1296 correlated aVMPs and, conversely, the aVMP *cg13246235* located near *PHACTR1* was associated with 853 associated *trans*-genes (Fig. 4b).

*Trans*-aVMPs encompassed two classes (instead of three in the complete set of aVMPs): 994 gain-aVMPs that, apart from increasing in variability with age, gained methylation and were hypomethylated in young individuals; and 822 loss-aVMPs that lost DNA methylation with age and were hypermethylated in young individuals (Figs. 1a and 4c). The genomic context of gain- and loss-aVMPs was markedly different. Gain-aVMPs were strongly enriched for CpG islands (CGIs) compared with loss-aVMPs (22.9-fold, *P <* 0.0001), while being depleted for non-CGI regions (Fig. 4d; 0.05-fold, *P <* 0.0001). Conversely, loss-aVMPs were highly enriched for non-CGI regions (19.0-fold, *P <* 0.0001; Fig. 4d). These results are in line with the fact that CGIs are commonly hypomethylated and hence can only gain DNA methylation, while non-CGI regions tend to be hypermethylated and preferentially lose DNA methylation. Furthermore, loss-aVMPs were generally found in active regions (*transcription flanking*, 4.3-fold enrichment, *P <* 0.01), but also in *weak polycomb repressed* regions (3.7-fold enrichment, *P <* 0.0001) (Fig. 4e). Gain-aVMPs were overrepresented in repressed and bivalent regions and particularly in *PcG repressed* regions (11.5-fold enriched, *P <* 0.0001; Fig. 4e). The enrichment analysis yielded similar results when gain- and loss-aVMPs were evaluated separately: both types were enriched for polycomb repressed regions and gain-aVMPs were enriched for bivalent domains. Bivalent domains have been associated with an enrichment for linear age-related DNA methylation changes [8].

Next, we investigated whether the age-related gain and loss of methylation at the *trans*-aVMPs extended beyond blood using publically available datasets. Gain-aVMPs showed a similar or even larger change in average methylation with age in, for example, colon (91.2 % concordant direction, *P* < 0.0001), lung (89.6 %, *P* < 0.0001), skin (87.0–93.3 %, *P* < 0.0001) and SAT (89.0 %, *P* < 0.0001) (Additional file 9). For loss-aDMPs, evidence for age-related changes in the same direction was found in lung (75.0 %, *P* < 0.0001), colon (70.9 %, *P* < 0.0001), and skin (56.3–66.1 %, *P* < 0.01). Within skin [34], age-related DNA methylation changes were most pronounced in the epidermis (compared with dermis), with the strongest effect sizes in sun-exposed epidermis samples. In view of the assumed commonalities between ageing and cancer, we investigated whether the changes also extend to different tumors given that gain of DNA methylation at CGIs is a hallmark of tumor biology [35]. Gain-aVMPs were found to show relative hypermethylation and loss-aVMPs hypomethylation, respectively, across various cancer types, including blood, bladder, colon, and lung cancer (*P* < 0.0001; Fig. 4f), which suggests a striking similarity between age-affected and cancer methylomes, in line with previous observations for aDMPs [36]. Taken together, aVMPs are largely driven by tissue-independent factors, but these changes may be accelerated by external influences (like sun exposure) and tumorigenesis.

### aVMP-associated *trans*-genes linked to DNA damage and apoptosis

**Fig. 5:**
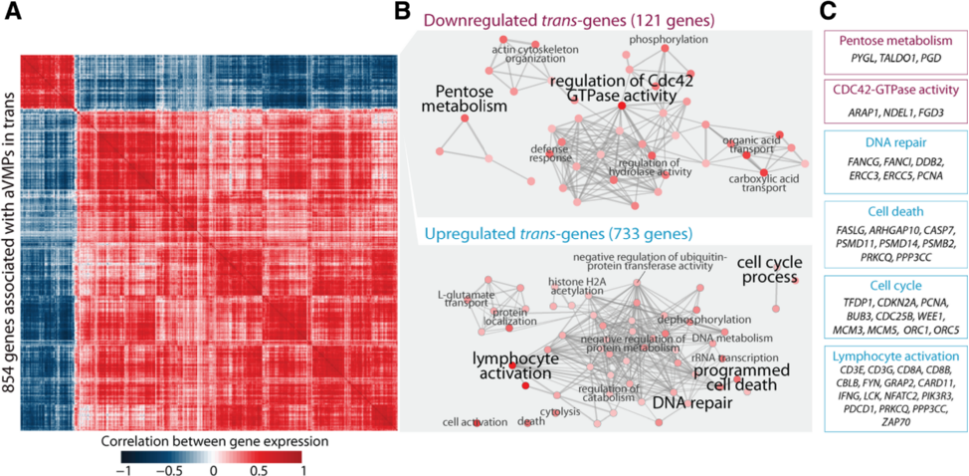
Annotation of genes associated with aVMP methylation *in trans*. **a** Correlation between genes associated with aVMP methylation *in trans*. **b** Enrichment of *trans*-genes in GO terms. **c** Examples of genes identified in each GO category

The expression of genes associated with aVMPs *in trans* unambiguously clustered into two highly correlated gene sets (Fig. 5a). An age-affected methylome was associated with down-regulated expression of *trans*-genes in a cluster of 121 genes and with up-regulated expression of a larger cluster of 733 genes. GO analysis showed that down-regulated genes are involved in various intra-cellular metabolic pathways, including pentose metabolism and regulation of *CDC42* GTPase activity (Fig. 5b), of which the former remained significant when using a permutation-based enrichment test (Additional file 10). Key genes in the pentose metabolism process whose expression was associated with aVMP methylation included *PYGL*, *TALDO1*, and *PGD* (Fig. 5b, c). Up-regulated *trans*-genes were intimately involved in apoptosis, cell cycle, DNA repair, and lymphocyte activation (Fig. 5b) and these enrichments were also observed using the permutation-based enrichment test (20,000 permutations; Additional file 10). The process unifying these pathways might be the cell damage response encompassing the upregulation of DNA repair, cell cycle changes, and upregulated apoptosis. In each category, key genes were identified, including the *ERCC* genes (DNA repair), *CDKN2A* (encoding the *INK4A*/*ARF* locus), *BUB3* (checkpoint), and *CASP7* (apoptosis) (Fig. 5c). Of note, a small proportion of the identified genes were previously found to be correlated with chronological age in 14,983 samples (163 genes, 19.1 %; Additional file 7b) [37], illustrating that the *trans*-genes we identified here represent, to a large extent, a different phenomenon. In summary, aVMP DNA methylation is associated with the downregulation of expression of genes in intra-cellular metabolism pathways and the upregulation of expression of genes in ageing pathways.

## Discussion

In a large-scale analysis we discovered and validated 6366 age-related variably methylated positions (aVMPs). These aVMPs were found to occur in three classes: gain-aVMPs that increase in variance and average DNA methylation with age, loss-aVMPs that increase in variance but decrease in average, and constant-aVMPs that increase in variance but with a constant average. aVMPs accrue in repressed regions that are characterized by both the PcG-deposited histone mark H3K27me3 and the binding site of EZH2, a component of PRC2. While aVMPs were commonly associated with the expression of (neuro)developmental genes *in cis*, they were linked to transcriptional activity of genes *in trans* that have a key role in well-established ageing pathways such as intracellular metabolism, apoptosis, and DNA damage response. Of interest, tumors were found to accumulate DNA methylation changes at CpG sites of aVMPs, thus supporting the long-standing notion that ageing and cancer are in part driven by common mechanisms [2].

Our data show that the genomic regions accumulating variability in ageing populations are highly specific and reproducible. Hence, although the increase in variability may have a stochastic component, the regions affected by this phenomenon are well-defined and not stochastic. aVMPs were discovered in whole blood samples (corrected for blood cell composition) and validated in independent whole blood and purified blood cell type (i.e., monocytes) samples. The results are thus unlikely to be driven by age-related changes in blood cell composition. Furthermore, we compared the DNA methylation of aVMPs to the DNA methylation of 19 blood cell subtypes and showed that the DNA methylation is highly concordant and, conversely, CpGs known to be differentially methylated between blood cell subtypes were not overrepresented among aVMPs. To our knowledge, our analyses together represent the most comprehensive validation of whole blood-discovered differential DNA methylation to date. Further support for the involvement of a cell type-independent phenomenon is the observation that aVMP methylation was associated with *in cis* and *in trans* expression of genes that function in developmental and ageing-related cellular processes, respectively, instead of immune pathways. To definitively confirm the cross-tissue occurrence of aVMPs, genome-wide DNA methylation data sets for internal tissues are required that have a similarly large size as those currently available for blood samples. Finally, aVMPs associated with gene expression *in trans* discovered in blood displayed a similar pattern of gain and loss of methylation with age across a series of tissues.

Importantly, our data indicate that aVMPs constitute a class of CpGs displaying age-related changes in the level of DNA methylation that is distinct from aDMPs. Known aDMPs rarely were aVMPs (2.6 % of 7477 previously reported aDMPs [7, 8, 14, 16, 18, 25]) and, in contrast to aDMPs, aVMPs showed striking associations with gene expression. Hence, aVMPs are not driven by changes in mean towards a methylation fraction of 0.5 that, in principle, could lead to an increased variance. This mathematical effect that cannot, however, be completely ruled out for all individual aVMPs.

aVMPs preferentially occur in repressed regions marked by PcG repression, namely the PcG binding site of EZH2 and repressive histone marks (H3K27me3). The integration of methylome with transcriptome data revealed that, in contrast to aDMP methylation, aVMP methylation is commonly associated with gene expression. Genes associated with aVMPs *in cis* frequently had a (neuro)developmental role exemplified by the *HOX* gene clusters, the protocadherin gene cluster, *TBX* genes, and *ZIC* genes. aVMPs reported here share the overrepresentation at PRC2-controlled regions with a subset of previously reported aDMPs [7, 8, 11, 12, 17, 36, 38], regions undergoing differential methylation observed in long-term cultured human senescent mesenchymal stem cells [39], various cancer types [7, 36], and, finally, regions displaying differential methylation in vitro after oxidative stress-induced DNA damage [40]. The latter study is of particular relevance to the interpretation of our results since it showed that oxidative DNA damage leads to PRC2 recruitment to sites with DNA damage [40] and results in translocation of DNA methyltransferases and EZH2 from CpG-poor regions to CG-rich regions, which in turn leads to hypermethylation of CGIs and hypomethylation at CpG-poor regions [40, 41]. We observed that aVMP methylation is frequently and strongly associated with the expression of PRC2 components *in trans*. Moreover, aVMPs were characterized by a gain of methylation at CGIs and a loss of methylation at CpG-poor regions. Our data highlight the potentially important role of altered PcG regulation in ageing.

Intriguingly, associations of aVMP methylation with gene expression *in trans* were not limited to PRC2 genes but extended to genes known to play a role in ageing. In older individuals who had an aged DNA methylation profile as compared with young individuals, we observed a downregulation of genes involved in metabolism. The upregulation of ageing pathways, as observed in old individuals with an aged methylome, has been reported previously in hematopoietic stem cells in mice and humans, for which macromolecular or DNA damage may be the driving force [42, 43]. Of note, many of the *trans*-genes we identified are involved in the DNA damage response and are frequently mutated in various cancers, including *CDKN2A*, *DNMT3A*, and *TP53* [44]. Hence, genomic stress, due either to hyperproliferation or DNA damage, may drive upregulation of well-established ageing pathways, downregulation of intra-cellular metabolism, and altered regulation by PcG proteins associated with increased variability of DNA methylation.

## Conclusions

In contrast to aDMPs, aVMPs show a striking variability in DNA methylation at higher ages. Two individuals of the same age may display highly distinct methylation patterns across aVMPs, where one of them may have a DNA methylation profile at aVMPs that is similar to that of young individuals. Therefore, aVMPs fulfill a primary prerequisite for a biomarker of biological age [26]. Further studies are required to establish whether aVMP-based methylation profiles mark health status and predict mortality. Finally, our study shows that large-scale integrative genomics studies are an effective approach toward the identification of fundamental processes involved in ageing and are complementary to experimental work in model organisms.

## Methods

### Data

DNA methylation data and RNA-seq data were generated within the Biobank-based Integrative Omics Studies Consortium (BIOS Consortium; Additional file 11) [45, 46]. Discovery data generated within the BIOS consortium are available from the European Genome-phenome Archive (EGA) under accession number EGAC00001000277. The data comprise six Dutch biobanks: Cohort on Diabetes and Atherosclerosis Maastricht (CODAM) [47], LifeLines (LL) [48], Leiden Longevity Study (LLS) [49], Netherlands Twin Registry (NTR) [50], Rotterdam Study (RS) [51], and the Prospective ALS Study Netherlands (PAN) [52]. A random co-twin per twin pair from the Netherlands Twin Registry was included in the current dataset to restrict our analysis to unrelated individuals. Briefly, 500 ng of genomic DNA was bisulfite converted using the EZ DNA Methylation kit (Zymo Research, Irvine, CA, USA) and hybridized on Illumina Infinium 450 arrays according to the manufacturer’s protocols and signal intensities measured using an Illumina iScan BeadChip scanner. Sample identity of DNA methylation and expression data was confirmed with genotype data using *MixupMapper* [53]. Quality control (QC) on the DNA methylation data was performed using the R package *MethylAid* [54]. Out of the 3391 samples, 3296 samples passed QC. Data were normalized using functional normalization implemented in the R package *minfi* using five principal components [55]. Ambiguously mapped probes [56], probes with a high detection *P* value (>0.01), probes with a low bead count (<3 beads), and probes with a low success rate (missing in >95 % of the samples) were set to missing. Probes mapping to chromosomes X and Y were excluded from all analyses. Residual batch effects were removed using Combat as implemented in the R package SVA, with biobank as batch and gender and age as outcome variables [57].

RNA-seq comprised 2044 expression profiles for which also DNA methylation data were available. RNA libraries were prepared using the Illumina’s Truseq version 2 library preparation kit and paired-end sequenced of 2 × 50 bp using Illumina’s Hiseq2000. Using Illumina’s *CASAVA*, read sets per sample were generated and only reads passing the Chastity Filter were used for further processing. Using *FastQC20* (v0.10.1) [58] initial QC was performed. Adapters were removed using *cutadapt21* (v1.1) [59]. Using *Sickle22* (v1.2) [60], low quality ends of the reads were trimmed (minimum length 25, minimum quality 20). Reads that passed QC were aligned to human genome build *hg19* using STAR23 (v2.3.125) [61] and gene quantifications were based on Ensembl version 71. Gene counts were normalized for GC content and gene length using the R package *cqn* [62]. Associations between gene expression and DNA methylation were performed using *voom*-transformed values [63]. For graphical purposes normalized counts were transformed to RPKM values. Generation of methylome and transcriptome data was performed by the Human Genotyping facility (HugeF) of the ErasmusMC (the Netherlands, http://www.glimdna.org/).

Cell count data (neutrophils, lymphocytes, monocytes, eosinophils, and basophils) were available for the majority of the samples (>60 %). A prediction model for blood cell composition was fitted using a subset of the data for which cell counts were available using a multivariate partial-least-squares model (including age and gender) on the normalized DNA methylation data. Using the fitted model, the cell fractions were imputed for all samples and only these fractions were used in all analyses. Documentation and code are available in the R package *wbccPredictor* (available from https://github.com/mvaniterson/wbccPredictor). Cell composition in the whole blood validation [25] dataset was also imputed using the described method. For the monocyte validation dataset, data on purity of the isolated cells was obtained from the Gene Expression Omnibus (GEO). To further confirm our results with the blood cell subtypes described by Houseman et al. [30], we used the implementation in the R package *minfi* [64] to impute the cell fractions of CD8^+^ T cells, CD4^+^ T cells, NK cells, B cells, and granulocytes.

Validation of aVMPs was performed in two external datasets, whole blood and monocyte datasets (Additional file 12). For the whole blood data, IDAT files used in Hannum et al*.* [25] were kindly provided by the authors. Data underwent the same quality and normalization procedure as used above. After quality control, 643 samples were used in subsequent samples. For the monocyte data, normalized data were obtained from the GEO [65] (accession number GSE56046 [17]). Raw DNA methylation data (Level 1; IDAT files) of healthy tissues and cancerous tissues were obtained from The Cancer Genome Atlas (TCGA Research Network, http://cancergenome.nih.gov; Additional file 12) and underwent QC and normalization equal to the discovery data. Normalized DNA methylation data for subcutaneous fat were obtained from the ArrayExpress [66] (accession number E-MTAB-1866 [67]). Normalized dermis and epidermis DNA methylation data were downloaded from GEO under accession number GSE51954 [34]. Normalized data for cytogenetic normal acute myeloid leukemia (CN-AML) and healthy bone marrow CD34+ cells were downloaded from GEO under accession number GSE58477 [68].

### Shannon entropy and aVMPs

All analyses were performed on the normalized whole dataset consisting of all biobanks together. To calculate the Shannon entropy, DNA methylation data were corrected for age, cell composition, and gender. Shannon entropy was calculated using a previously described method for DNA methylation data [25].

aVMPs were identified by using the Breusch–Pagan test for heteroscedasticity [69]. First, average change in age, blood cell composition, and gender were regressed out. Next, squared residuals were tested for an association with age with correction for blood cell composition and gender. aVMPs were defined as CpGs that showed a significant association between squared residuals and age with a Bonferroni corrected *P* value ≤0.05. Moreover, aVMPs were only selected if the increased variability with age was larger than 5 % per 10 years. aVMPs were further subdivided into three classes based on a clustering analysis: gain-aVMPs, loss-aVMPs, and constant-aVMPs. Gain-aVMPs were defined as CpGs that were hypomethylated at young age but where the change in average DNA methylation was positive (Fig. 1d middle panel). Loss-aVMPs were defined as CpGs that were hypermethylated at young age with a negative change in average DNA methylation with age (Fig. 1d bottom panel). Constant-aVMPs were defined as CpGs that were intermediately methylated and did not show a change in average but only in variance (Fig. 1d top panel).

For the association between DNA methylation of aVMPs and gene expression *in cis*, we only considered genes within 500 kb of the aVMP and we adjusted for gender and blood cell composition. The *P* value was corrected for multiple testing using the Bonferroni method for the number of genes tested *in cis* (*P*_bonf_ ≤ 0.05). Next, the most significant associated gene (if any) was selected. Finally, the *P* value of each of these aVMP-gene pairs was corrected using the false discovery rate (FDR) method (*P*_FDR_ ≤ 0.05) and only significant pairs were used [70].

*In trans*, we only evaluated aVMP and gene pairs that were >5 Mb away from each other on the same or on a different chromosome. *P* values for *trans*-associations were FDR corrected (*P*_FDR_ ≤ 0.05 considered significant). Genes whose gene expression was associated with less than 5 % of all aVMPs (≤318 aVMPs, 6366) *in trans* were discarded.

Z-scores (compared to young) were calculated as a measure of directionality and magnitude of aberrant methylation of an aVMP within an individual:

$$ {Z}_{i,j}=\frac{\left({M}_{i,j}-\overline{M_{y,i}}\right)}{\sigma_{y,i}} $$

where *Z*_*i,j*_ is the Z-score of the *j*^*th*^ individual and *i*^*th*^ aVMP, *M*_*i,j*_ the DNA methylation of the *j*^*th*^ individual and *i*^*th*^ aVMP, $\overline{M_{y,i}}$ the average DNA methylation in young individuals (<30 years) of the *i*^*th*^ aVMP, and *σ*_*y*,*i*_ the standard deviation in young individuals of the *i*^*th*^ aVMP. All heatmaps were clustered based on Euclidian distance.

aVMPs were clustered to aVMRs using a method described before using default settings [33]. Genes were linked to aVMRs based on the nearest protein coding gene (Ensembl).

### Annotations and integration with external data

Chromatin state segments were downloaded from the Epigenomics Roadmap for different blood cell subtypes [32]. CpGs were annotated to different segments based on the most frequent occurring feature in the various blood cell subtypes. Transcription factor binding sites (ChIP-seq) were obtained from the ENCODE project for all tissue types available [71]. CGI-centric annotations were obtained from previous work [33]. Enrichments were expressed as odds ratio on a log2 scale (if applicable).

GO enrichments were performed using the default settings of DAVID. To reduce redundancy in the GO enrichment terms, we used REVIGO with default settings [72]. Networks were plotted using Cytoscape version 3.2.1 [73]. The enrichment for gene sets was further verified with the gene set enrichment function (gseGO, biological processes) in the R package *clusterProfiler* [74] based on the fold change of the association between DNA methylation and gene expression *in trans* and 20,000 permutations.

To compare aVMPs in healthy tissues other than blood a linear model was fitted between DNA methylation and age for colon (TCGA), lung (TCGA), kidney (TCGA), bladder (TCGA), thyroid (TCGA), subcutaneous fat (GEO) [67] and skin (GEO) [34]. To compare healthy and cancerous tissues, data for the *trans-*aVMPs were obtained in CN-AML versus bone marrow CD34^+^ cells (GEO) [68], bladder urothelial carcinoma versus healthy bladder (TCGA), colon adenocarcinoma versus healthy colon (TCGA), kidney renal clear squamous cell carcinoma versus healthy kidney (TCGA), lung adenocarcinoma versus healthy lung (TCGA), and thyroid carcinoma versus healthy thyroid (TCGA).

Analyses were performed using *R* statistics, version 3.1.2. Figures were produced using *ggplot2* [75], the Perl version of Circos (v0.67-7) [76] and Gviz [77].

## Additional files

Additional file 1: Data characteristics and characteristics of aVMPs. a Sample description per biobank. b Distribution of age in the samples used. c Example of an age-independent CpG site where the DNA methylation does not change in age or variance with age near *NOC2L*. d Example of an aDMP, with an average change in DNA methylation but not variance near *BMI1*. e Overlap between aVMPs and frequently identified aDMPs and CpGs in Horvath’s ageing clock [24]. f Heatmap of Z-score of individuals (*columns*) versus young individuals (<30 years) of all 6366 aVMPs (*rows*). g Correlation between individuals (*y-axis*) for each of the age groups in our study (*x-axis*). The *blue curve* is a loess smoothed curve. (TIF 4010 kb) Additional file 2: List of validated aVMPs and their genomic location (hg19). (XLSX 234 kb) Additional file 3: Comparison of whole blood DNA methylation (discovery data) to blood sub-cell type DNA methylation. The two plots below each blood cell type are based on aVMPs (*left*) and as a control the 600 CpGs from Jaffe and Irizarry [31] (*right*), of which it is know that the DNA methylation between cell types is known to be different. Percentages were obtained from https://www.stemcell.com/media/files/wallchart/WA10006-Frequencies_Cell_Types_Human_Peripheral_Blood.pdf. (TIF 6010 kb) Additional file 4: Enrichment of aVMPs for histone modifications and relation with expression *in cis*. a Enrichment (odds ratio, *y-axis*) for histone modifications of aVMPs (*x-axis*). b Enrichment (odds ratio, *y-axis*) of gain- and loss-aVMPs in chromatin state segments (*x-axis*). c Two examples of associations between aVMPs and gene expression of *PCDHB6* and *HOXC4*. (TIF 1170 kb) Additional file 5: Relation between DNA methylation and gene expression *in cis.* (XLSX 119 kb) Additional file 6: Identified aVMRs with nearest protein coding gene. (XLSX 19 kb) Additional file 7: a Correlation between DNA methylation and gene expression of PcG complex proteins. b Overlap between genes associated *in cis* and *in trans* with the transcriptome clock [37]. (TIF 531 kb) Additional file 8: Relationship between DNA methylation and gene expression *in trans*. (XLSX 41 kb) Additional file 9: Slope of average DNA methylation of aVMPs in various healthy tissues. (TIF 559 kb) Additional file 10: Enrichment of identified *trans*-genes in GO terms based on 20 k permutations. (XLSX 104 kb) Additional file 11: BIOS Consortium (Biobank-based Integrative Omics Study). (PDF 68 kb) Additional file 12: Description of external datasets used. (PDF 79 kb)
