# Supplementary figures and images for: Age-related accrual of methylomic variability is linked to fundamental ageing mechanisms
Source: Genome Biol. 2016 Sep 22;17:191. doi: 10.1186/s13059-016-1053-6 (PMC5032245; doi:10.1186/s13059-016-1053-6)

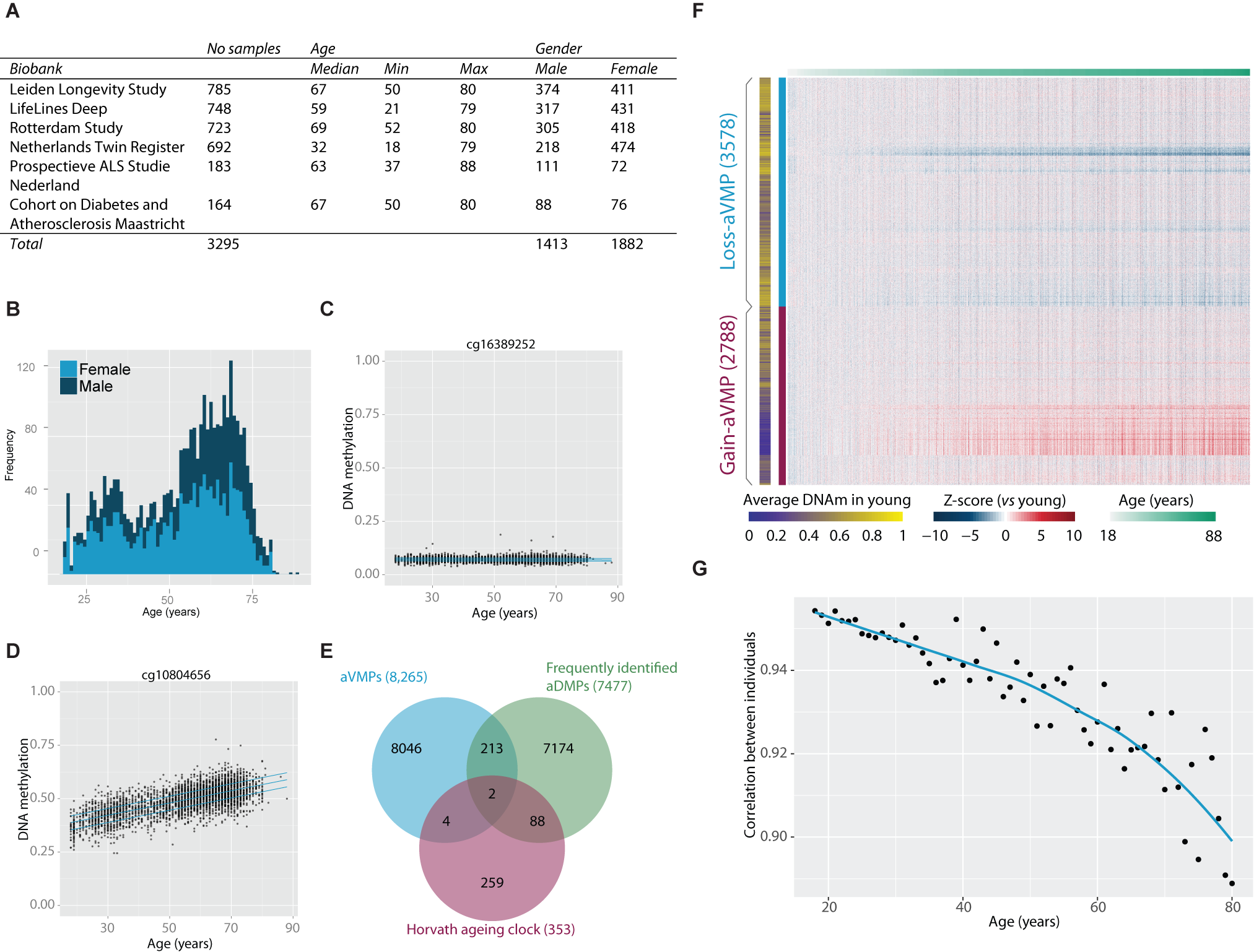

Supplement: Additional file 1: — Data characteristics and characteristics of aVMPs. a Sample description per biobank. b Distribution of age in the samples used. c Example of an age-independent CpG site where the DNA methylation does not change in age or variance with age near NOC2L. d Example of an aDMP, with an average change in DNA methylation but not variance near BMI1. e Overlap between aVMPs and frequently identified aDMPs and CpGs in Horvath’s ageing clock [24]. f Heatmap of Z-score of individuals (columns) versus young individuals (<30 years) of all 6366 aVMPs (rows). g Correlation between individuals (y-axis) for each of the age groups in our study (x-axis). The blue curve is a loess smoothed curve. (TIF 4010 kb) [file 13059_2016_1053_MOESM1_ESM.tif]

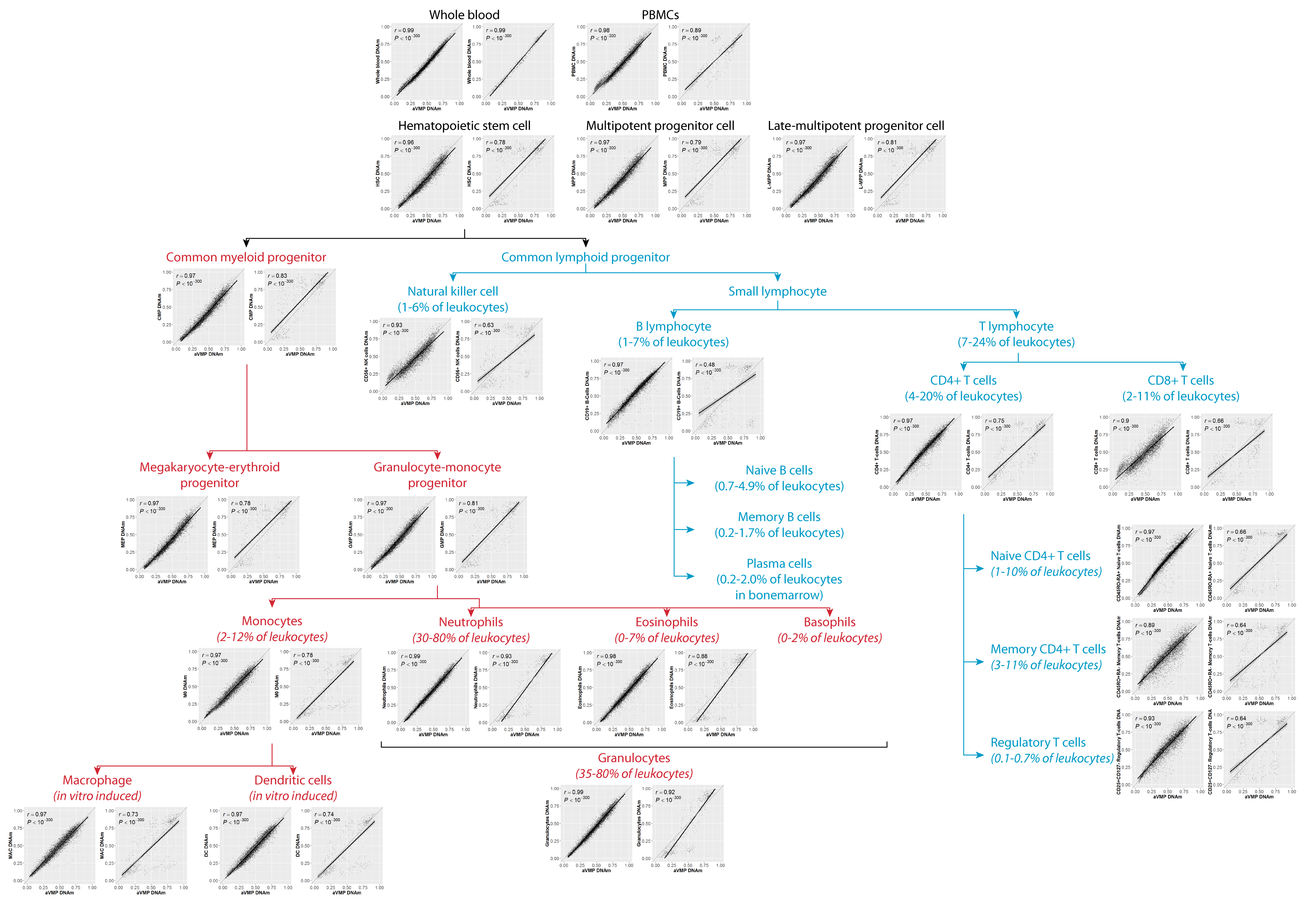

Supplement: Additional file 3: — Comparison of whole blood DNA methylation (discovery data) to blood sub-cell type DNA methylation. The two plots below each blood cell type are based on aVMPs (left) and as a control the 600 CpGs from Jaffe and Irizarry [31] (right), of which it is know that the DNA methylation between cell types is known to be different. Percentages were obtained from https://www.stemcell.com/media/files/wallchart/WA10006-Frequencies_Cell_Types_Human_Peripheral_Blood.pdf. (TIF 6010 kb) [file 13059_2016_1053_MOESM3_ESM.tif]

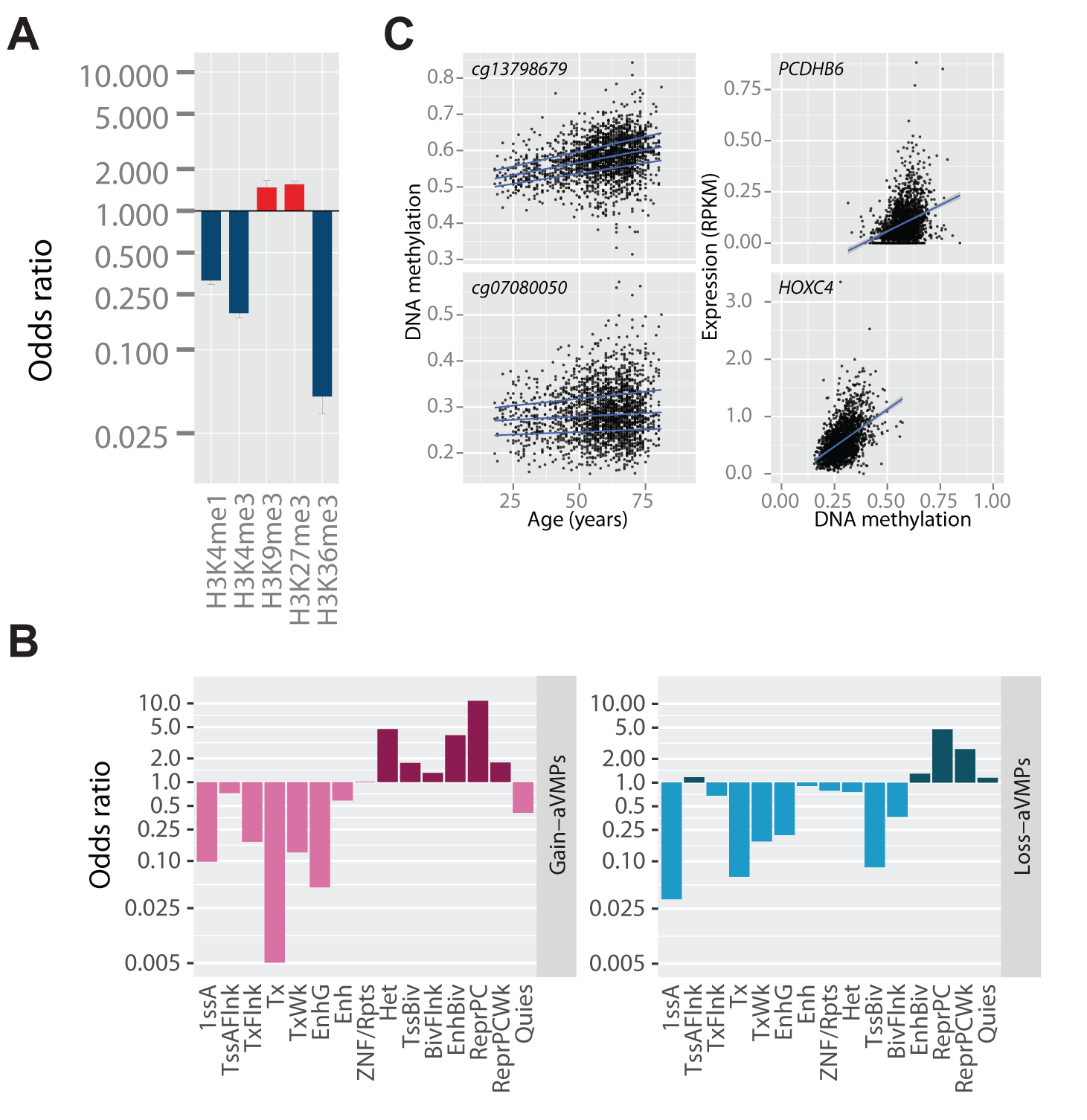

Supplement: Additional file 4: — Enrichment of aVMPs for histone modifications and relation with expression in cis. a Enrichment (odds ratio, y-axis) for histone modifications of aVMPs (x-axis). b Enrichment (odds ratio, y-axis) of gain- and loss-aVMPs in chromatin state segments (x-axis). c Two examples of associations between aVMPs and gene expression of PCDHB6 and HOXC4. (TIF 1170 kb) [file 13059_2016_1053_MOESM4_ESM.tif]

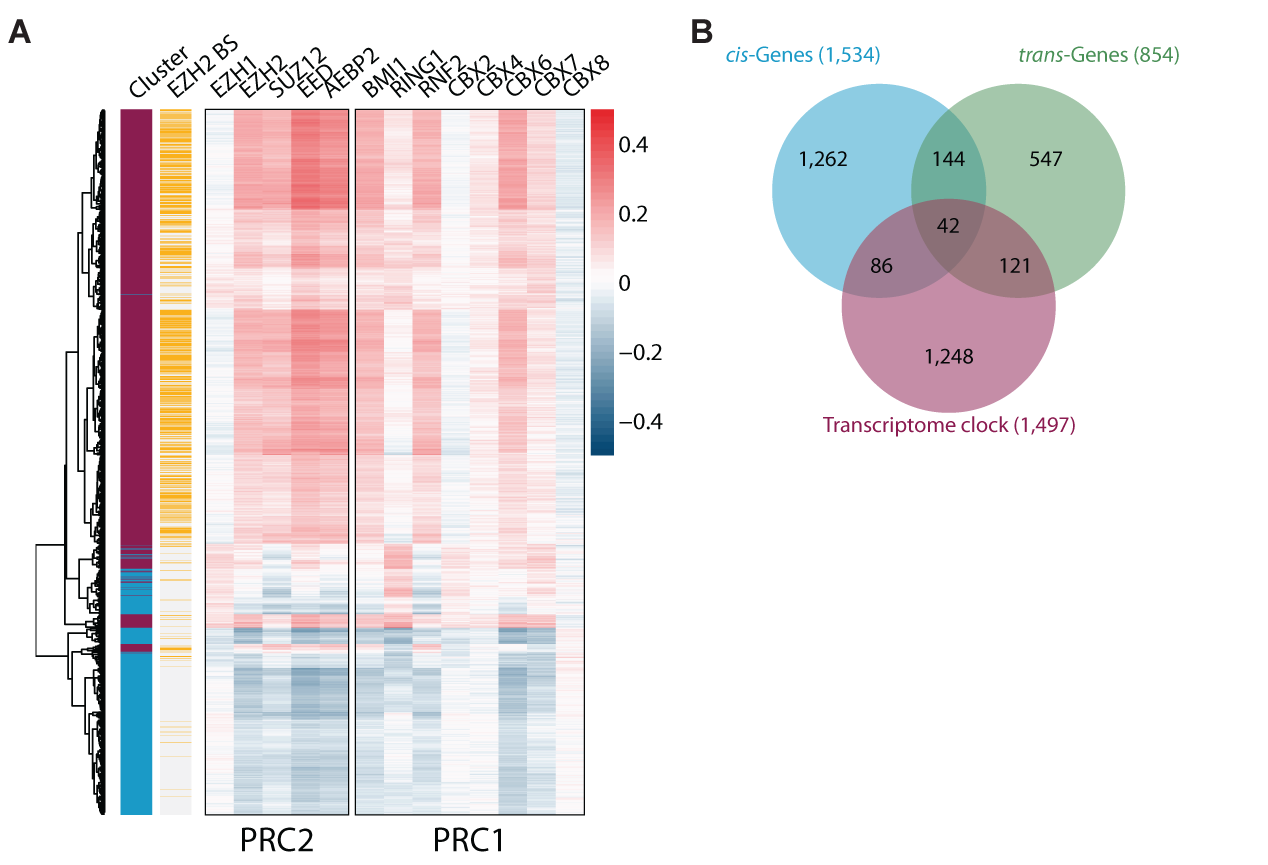

Supplement: Additional file 7: — a Correlation between DNA methylation and gene expression of PcG complex proteins. b Overlap between genes associated in cis and in trans with the transcriptome clock [37]. (TIF 531 kb) [file 13059_2016_1053_MOESM7_ESM.tif]

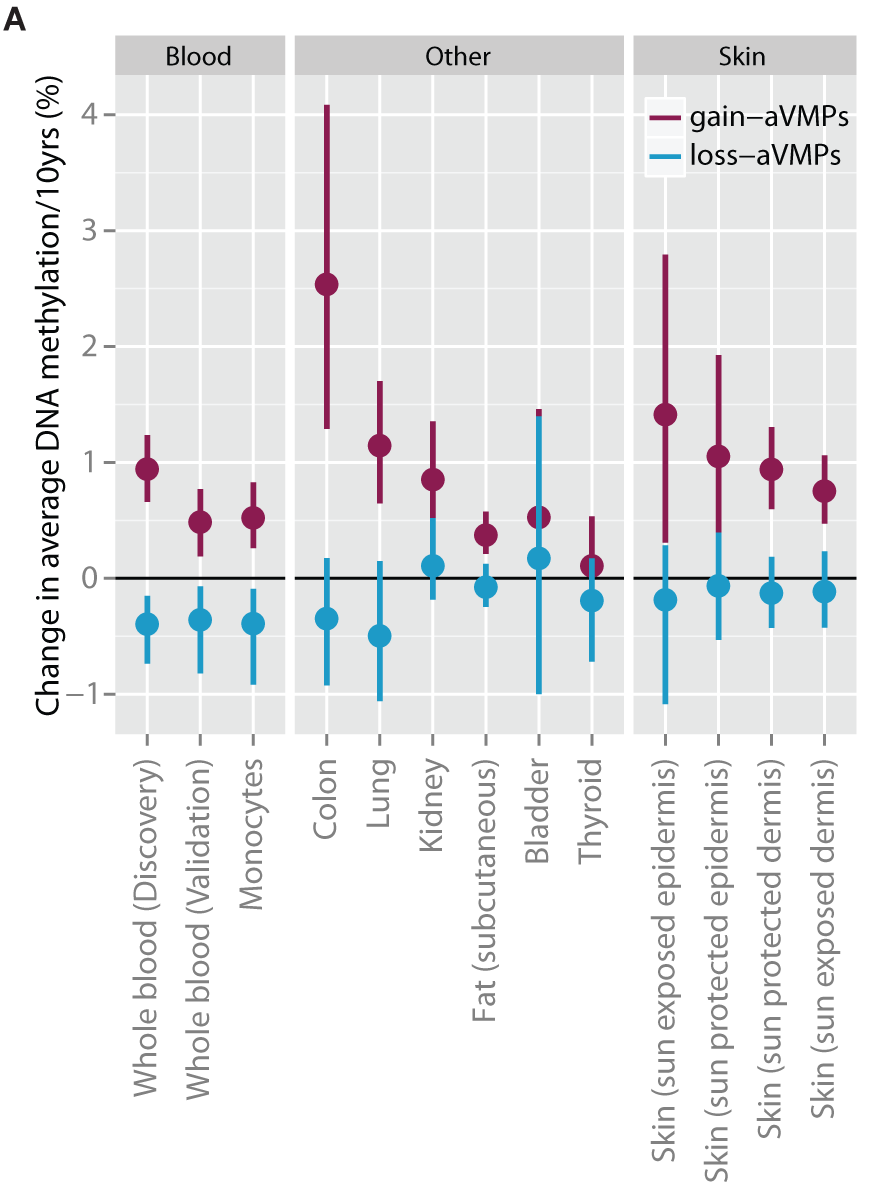

Supplement: Additional file 9: — Slope of average DNA methylation of aVMPs in various healthy tissues. (TIF 559 kb) [file 13059_2016_1053_MOESM9_ESM.tif]
