# Supplementary material for: Age-related accrual of methylomic variability is linked to fundamental ageing mechanisms
Source: Genome Biol. 2016 Sep 22;17:191. doi: 10.1186/s13059-016-1053-6 (PMC5032245; doi:10.1186/s13059-016-1053-6)
Supplement: Additional file 12: — Description of external datasets used. (PDF 79 kb) [file 13059_2016_1053_MOESM12_ESM.pdf]

Tissue	No of samples	Age			Accession	Reference
		Median	Min	Max		
Whole blood	643	65	20	102	GSE40279	(Hannum et al., 2013)
Monocytes (CD14+)	1202	60	44	83	GSE56046	(Reynolds et al., 2014)
Tissues						
Bladder	14	70.5	48	84	TCGA	TCGA
Colon	38	72	40	90	TCGA	TCGA
Kidney	160	63	38	90	TCGA	TCGA
Lung	32	64.5	42	86	TCGA	TCGA
Thyroid	56	42	15	81	TCGA	TCGA
Normal bone marrow progenitor cells	10	-	-	-	GSE58477	(Qu et al., 2014)
Skin (dermis - exposed & unexposed)	20	49.5	20	90	GSE51954	(Vandiver et al., 2015)
Skin (epidermis - exposed & unexposed)	19	65	20	90	GSE51954	(Vandiver et al., 2015)
Subcutaneous fat	648	60	39	85	E-MTAB-1866	(Grundberg et al., 2012)
Tumors						
Bladder urothelial carcinoma (BLCA)	250	68	34	90	TCGA	TCGA
Colon adenocarcinoma (COAD)	296	68	31	90	TCGA	TCGA
Kidney renal clear cell carcinoma (KIRC)	307	61	26	90	TCGA	TCGA
Lung adenocarcinoma (LUAD)	400	65	33	86	TCGA	TCGA
Thyroid carcinoma (TCHA)	469	46	15	89	TCGA	TCGA
Acute myeloid leukemia (AML)	62	53	27	77	GSE58477	(Qu et al., 2014)

Grundberg, E., Small, K.S., Hedman, Å.K., Nica, A.C., Buil, A., Keildson, S., Bell, J.T., Yang, T.-P., Meduri, E., and Barrett, A. (2012). Mapping cis-and trans-regulatory effects across multiple tissues in twins. Nature genetics *44*, 1084-1089.

Hannum, G., Guinney, J., Zhao, L., Zhang, L., Hughes, G., Sadda, S., Klotzle, B., Bibikova, M., Fan, J.B., Gao, Y.*, et al.* (2013). Genome-wide methylation profiles reveal quantitative views of human aging rates. Molecular cell *49*, 359-367.

Qu, Y., Lennartsson, A., Gaidzik, V.I., Deneberg, S., Karimi, M., Bengtzén, S., Höglund, M., Bullinger, L., Döhner, K., and Lehmann, S. (2014). Differential methylation in CN-AML preferentially targets non-CGI regions and is dictated by DNMT3A mutational status and associated with predominant hypomethylation of HOX genes. Epigenetics *9*, 1108-1119.

Reynolds, L.M., Taylor, J.R., Ding, J., Lohman, K., Johnson, C., Siscovick, D., Burke, G., Post, W., Shea, S., Jacobs Jr, D.R.*, et al.* (2014). Age-related variations in the methylome associated with gene expression in human monocytes and T cells. Nat Commun *5*.

Vandiver, A.R., Irizarry, R.A., Hansen, K.D., Garza, L.A., Runarsson, A., Li, X., Chien, A.L., Wang, T.S., Leung, S.G., and Kang, S. (2015). Age and sun exposure-related widespread genomic blocks of hypomethylation in nonmalignant skin. Genome biology *16*, 80.
